# Systemic Transplantation of Human Adipose Tissue-Derived Mesenchymal Stem Cells for the Regeneration of Irradiation-Induced Salivary Gland Damage

**DOI:** 10.1371/journal.pone.0071167

**Published:** 2013-08-09

**Authors:** Jae-Yol Lim, Jeong Chan Ra, Il Seob Shin, Yun Ho Jang, Hye-Young An, Jeong-Seok Choi, Woo Cheol Kim, Young-Mo Kim

**Affiliations:** 1 Department of Otorhinolaryngology - Head and Neck Surgery, Inha University School of Medicine, Incheon, Republic of Korea; 2 Stem cell research center, RNL Bio Co., Ltd., Seoul, Republic of Korea; 3 Department of Radiation Oncology, Inha University School of Medicine, Incheon, Republic of Korea; Institut Gustave Roussy, France

## Abstract

**Objectives:**

Cell-based therapy has been reported to repair or restore damaged salivary gland (SG) tissue after irradiation. This study was aimed at determining whether systemic administration of human adipose-derived mesenchymal stem cells (hAdMSCs) can ameliorate radiation-induced SG damage.

**Methods:**

hAdMSCs (1×10^6^) were administered through a tail vein of C3H mice immediately after local irradiation, and then this infusion was repeated once a week for 3 consecutive weeks. At 12 weeks after irradiation, functional evaluations were conducted by measuring salivary flow rates (SFRs) and salivation lag times, and histopathologic and immunofluorescence histochemistry studies were performed to assay microstructural changes, apoptosis, and proliferation indices. The engraftment and *in vivo* differentiation of infused hAdMSCs were also investigated, and the transdifferentiation of hAdMSCs into amylase-producing SG epithelial cells (SGCs) was observed *in vitro* using a co-culture system.

**Results:**

The systemic administration of hAdMSCs exhibited improved SFRs at 12 weeks after irradiation. hAdMSC-transplanted SGs showed fewer damaged and atrophied acinar cells and higher mucin and amylase production levels than untreated irradiated SGs. Immunofluorescence TUNEL assays revealed fewer apoptotic cells in the hAdMSC group than in the untreated group. Infused hAdMSCs were detected in transplanted SGs at 4 weeks after irradiation and some cells were found to have differentiated into SGCs. *In vitro,* a low number of co-cultured hAdMSCs (13%–18%) were observed to transdifferentiate into SGCs.

**Conclusion:**

The findings of this study indicate that hAdMSCs have the potential to protect against irradiation-induced cell loss and to transdifferentiate into SGCs, and suggest that hAdMSC administration should be viewed as a candidate therapy for the treatment of radiation-induced SG damage.

## Introduction

Salivary hypofunction with its subjective symptom of dry mouth (xerostomia) is the most significant long-term complication of radiotherapy for the treatment of head and neck cancers. Each year, 500,000 new cases of head and neck cancer develop worldwide and the majority of advanced cases require radiotherapy with or without chemotherapy as a primary or adjuvant treatment following surgery. A systematic review by Jensen et al. revealed that the prevalence of xerostomia ranges from 74 to 85% after all radiation therapies for head and neck cancer, and that salivary secretion and xerostomia showed incomplete improvements, even after parotid-sparing intensity-modulated radiation therapy. [1].

Saliva is required for digestion, lubrication, oral homeostasis, and for protection against a variety of noxious materials and microorganisms, and salivary hypofunction resulting from radiation damage to the salivary glands (SG) can cause various life-disrupting side effects, such as, swallowing difficulties, taste loss, oral candidiasis, and dental caries. [2] Furthermore, hyposalivation may be an irreversible life-long condition and significantly affect quality of life. Nevertheless, no satisfactory therapy has been devised to treat salivary hypofunction, and current treatment strategies are confined to the minimization of SG radiation damage by parotid-sparing radiation delivery or conservative care based on the use of salivary substitutes and sialogogues. [3].

Interest in therapeutic strategies designed to repair and/or restore damaged SGs is increasing, and in the context of tissue engineering and regenerative medicine, these strategies include the re-implantation of autologous SG cells, [4] the implantation of engineered artificial SGs, [5] stem cell therapy, [6], [7] and gene therapy. [8] Bone-marrow-derived cells (BMCs) were recently proposed as potential candidates for the treatment of salivary hypofunction.[9]–[12] Adipose tissue-derived mesenchymal stem cells (AdMSCs) are another potent source of adult stem cells, and can be readily aspirated using a minimally invasive procedure and are relatively unaffected by donor age. In addition, adipose tissues contain higher densities of MSCs than bone marrow. [13] For these reasons, AdMSC based treatments for a variety of diseases have been investigated for use in the tissue engineering and regenerative medicine fields.

Stem cells have an inherent ability to mobilize to injured tissues, for example, adult BMCs intravenously delivered to rats after myocardial infarction homed to infarcted regions and improved ventricular function, whereas stem cells delivered to noninfarcted rats localized to bone marrow. [14] Lombaert *et al.* found that BMCs pretreated with a mobilizing agent, mobilized to irradiated glands, and ameliorated acinar cell loss and vascular damage. [10], [11] If safety concerns regarding the intravenous infusion of stem cells are met, they would provide a straightforward and minimally invasive means of delivery.

We undertook the present study to determine whether the systemic administration of human adipose-derived mesenchymal stem cells (hAdMSCs) could mobilize to damaged SGs and ameliorate radiation-induced SG damage. In addition, we studied whether the systemic transplantation of hAdMSCs into the irradiated SGs could provide protection against radiation-induced apoptosis, increase SG regeneration, and transdifferentiate into salivary gland epithelial cells (SGCs) of endodermal lineage.

## Materials and Methods

### Ethics Statement

This study was approved by the Animal Ethics Committee of Inha University Hospital (Permit Number: 110829-110). Animals were cared for according to established institutional guidelines, and all efforts were made to minimize suffering. Surgeries were performed under anesthesia after xylazine (10 mg/kg) premedication and an intraperitoneal injection of ketamine (110 mg/kg), and all efforts were made to minimize suffering.

### Preparation of hAdMSCs

hAdMSCs were prepared from surplus banked stem cells under good manufacturing practice (GMP) conditions (RNL Bio, Seoul) and used as ‘anonymized’ materials. IRB approval for use of the cells was not required for the research. [15] In brief, human abdominal subcutaneous fat tissues were obtained by simple liposuction with informed consent from donors. Aspirated fat tissues were separated from fluids by centrifugation and digested with RNase (RNL Bio, Seoul) for 60 min at 37°C. The digested tissues were filtered through a 100 µm nylon sieve to remove cellular debris, and the pellets obtained by centrifugation were resuspended in RCME cell attachment medium (RNL BIO) containing 10% fetal bovine serum (FBS; Invitrogen, Carlsbad, CA) and cultured overnight at 37°C. After 24 h, the cell medium was changed to RKCM cell growth medium (RNL Bio) containing 5% FBS. When the cells reached 80–90% confluency, they were subcultured and expanded in RKCM. They were further characterized for MSC identification and cells in their third passage were used for experiments.

#### Flow cytometry

For cell surface phenotyping, undifferentiated hASC were stained with phycoerythrin (PE)- or fluorescein-isothiocyanate (FITC)-conjugated antibodies against human antigens CD29 (PE-555443), CD31 (FITC-555445), CD34 (PE-555822), CD44 (FITC-555478), CD45 (FITC-555482), CD73 (PE-550257), CD90 (PE-555596), CD105 (PE-560839), HLA-ABC (FITC-555552) and HLA-DR (FITC-555811) (all from BD Pharmingen, San Diego, CA) and analyzed by fluorescent-activated cell sorting using FACSCalibur and CellQuest software (BD Biosciences, San Jose, CA).

#### *In vitro* differentiation

The abilities of hAdMSCs to differentiate into the osteogenic, adipogenic, and chondrogenic lineage were investigated. Osteogenic or adipogenic differentiation was induced by culturing cells for 3 weeks in osteogenic or adipogenic medium (Miltenyi Biotec, Gladbach, Germany). Media were changed three times per week. The osteogenic differentiation of hAdMSCs was assessed by evaluating morphological changes and calcified extracellular matrix deposition as demonstrated by positive Alizarin red staining. Adipogenesis was measured by staining lipid droplets with Oil Red O. Cells cultured in standard cell culture medium were used as undifferentiated controls.

Chondrogenic differentiation was induced over 14 days in pellets maintained in polypropylene tubes in 50% fresh chondrogenic medium (Lonza, Walkersville, MD), which was replaced every third day. Chondrogenesis was evaluated by histological toluidine blue staining of pellet sections at Green Cross Co. (Yongin, Korea).

### Animal Model

Eight to nine-week-old female C3H mice weighing 18 ∼ 22 g were purchased from the Research Model Producing Center (Orient Bio, Gyeonggi-Do, Korea). Animals were maintained under conventional clean conditions, and provided with standard laboratory chow and sterilized water *ad libitum*.

To cause irradiation-induced salivary dysfunction, animals were firmly fixed in a plastic mold and irradiated with a 4 MV X-ray from a linear accelerator (Mevatron MD, Siemens Medical Laboratories Inc., Germany) using a single dose of 15 Gy at a focus-to-skin distance of 100 cm. Animals were locally irradiated in the head and neck region, including the salivary gland, with bodies shielded from the radiation field. The dosage required to induce hyposalivation without compromising general health was calculated as described previously. [16].

### Transplantation

Experimental animals were randomly divided into 3 groups of 20 mice, namely, a non-irradiated group (the normal control group), an irradiated/untreated group (the irradiated group), and a group that received hAdMSCs after irradiation (the hAdMSC group). Mice in the hAdMSC group received 1×10^6^ hAdMSCs through a tail vein immediately after irradiation, and this infusion was repeated weekly for 3 consecutive weeks (Fig. S1).

### Morphological and Functional Evaluations

#### Gland and body weights

Body weights were measured at 12 weeks after irradiation and followed by saliva collection and humane euthanasia. Submandibular glands were harvested and surrounding fat and connective tissues were removed. The weights of both submandibular glands were measured before fixation in 10% neutral formalin buffer and embedding in paraffin.

#### Salivary flow rates and lag times

Salivary secretory function was determined by measuring salivary flow rates (SFRs) and lag times at 12 weeks after irradiation. Saliva was collected from the floor of the mouth using a micropipette for 5 min after stimulation with an intraperitoneal injection with pilocarpine (2 mg/kg). Collected saliva was placed in pre-weighed 1.5 ml microcentrifuge tubes and SFR (µL/min) was calculated by dividing the weight (mg) of all saliva collected by the collection time (min) (saliva was assumed to have a specific gravity of 1 mg/mL). Salivary lag time was defined as time from stimulation to the beginning of saliva secretion.

### Histological and Immunohistochemical Evaluations of Changes in the Structure and Functionalities of Acinar Cells

After deparaffinization and rehydration, tissue sections were analyzed by Hematoxylin-Eosin (H-E) and Masson’s trichrome (MTC) staining. The functionalities of acinar cells were studied by measuring mucin production by Alcian Blue (AB) staining and amylase production by immunohistochemistry (described below) in SG tissues. The surface areas of mucin or amylase-containing acinar cells were determined by densitometry. Three sections were prepared for each gland and at least 3 fields per section were examined by two blinded investigators using Metamorph software (Molecular Devices Corporation, Sunnyvale, CA).

### Immunofluorescence Confocal Microscopy

#### Apoptosis

Apoptotic cells in submandibular glands were visualized using the Apoptag Plus Fluorescein In Situ Apoptosis Detection kit (Millipore, Bedford, MA), which uses terminal deoxynucleotidyl transferase dUTP nick-end labeling (TUNEL) to detect DNA cleavage and chromatin condensation. After deparaffinization and rehydration, slides were incubated with the TUNEL reaction mixture containing TdT enzyme for 1 h at 37°C, and then with anti-digoxigenin fluorescein for 30 min at room temperature. Nuclei were visualized using 4′,6-diamidino-2-phenylindole dihydrochloride (DAPI). Two blinded examiners independently counted numbers of apoptotic cells in 3 random fields per tissue section. At least 3 random tissue sections per gland were mounted on each slide.

#### Cell proliferation

To detect proliferating cells, tissue sections were stained with anti-proliferating cell nuclear antigen (PCNA, Cell Signaling, Beverly, MA). After deparaffinization and rehydration, tissue sections were treated with citrate buffer solution (9 mL of 0.1 M citric acid and 41 mL of 0.1 M sodium citrate in 450 mL distilled water) in a 600 W microwave and then allowed to cool to room temperature. Tissue sections were then incubated with primary mouse PCNA antibody overnight at 4°C, washed in PBS, blocked, and incubated with Alexa Fluor 555 conjugated anti-mouse IgG (Cell Signaling, Beverly, MA) for 1 h at room temperature. Two blinded independent examiners then counted numbers of apoptotic cells in three random fields per tissue section. At least three random tissue sections per gland were mounted on each slide.

#### *In vivo* engraftment and transdifferentiation

Fluorescent in situ hybridization (FISH) analysis was performed using human-specific Alu probes. Briefly, at least three random tissue sections per gland from ten mice were chosen for evaluation. Salivary tissue sections were deparaffinized and subjected to epitope retrieval in citrate buffer (pH 6.0).

Tissue section slides were then incubated with a fluorescein-labeled Alu probe (PR-1001-01; BioGenex, San Ramon, CA), denatured at 85°C for 10 minutes, and hybridized overnight at 37°C in moist chamber. After stringent washing, nonspecific binding was blocked with 1% BSA for 60 min at 37°C. Human Alu probe was detected using biotinylated anti-fluorescein antibody (10 µg/mL, Vector Laboratories, Burlingame, CA) followed by fluorescein (FITC) conjugated AffiniPure goat anti-Armenian hamster IgG (10 µg/mL, Jackson ImmnoResearch Labs, West Grove, PA).

Primary polyclonal anti-α-amylase (1∶100, Cell Signaling) antibodies were used to test the functionality of acinar cells and the differentiation of hAdMSCs into SGCs. Tissue sections were incubated with primary polyclonal anti-α-amylase antibodies overnight at 4°C and then washed 3 times with PBS. Slides were incubated at room temperature for 2 h with Alexa Fluor 555 conjugated anti-mouse IgG (Cell Signaling, Beverly, MA). Nuclei were visualized with DAPI. All experiments included a slide with no primary antibody as a negative control. Slides were visualized using a confocal laser scanning microscope (Olympus FV1000, Japan).

### *In vitro* Differentiation

#### Isolation of SGCs

SGCs were obtained from adult mice. In brief, submandibular glands were harvested from three 5-week-old male C3H mice, mechanically minced with a fine curved scissors, and enzymatically dissociated with 0.1% collagenase type II. After inactivating collagenase activity with 10% FBS, cell pellets were filtered through a 70 µm cell strainer, resuspended in medium containing 10% FBS and 1% antibiotics, and then cultured at 37°C in a 5% CO_2_ atmosphere in a humidified incubator. After 5 days of culture, nonadherent cells were discarded, and adherent cells were thoroughly washed twice with 10% PBS.

#### Co-culture of hAdMSCs and SGCs

To test for the generation of amylase producing cells among hAdMSCs *in vitro*, a co-culture system was used whereby isolated SGCs and hAdMSCs were cultured for 5 days in the two chambers of a Transwell system separated by a polycarbonate membrane (Transwell-Clear system 6-well plate; SPL Lifesciences, Pocheon, Korea). For co-culture experiments, hAdMSCs (2×10^4^ cells/6-well and 3×10^3^ cells/24-well) were cultured in the lower chamber and SGCs (2×10^4^ cell/6-well and 3×10^3^ cells/24-well) in the upper chamber (i.e., on the polyester membrane). The culture medium used was a low-glucose DMEM containing 1% antibiotics and 10% FBS. The polycarbonate membrane had 0.4 µm-sized pores, which allowed the passage of soluble factors between hAdMSCs and SGCs, but did not allow cells to migrate from one chamber to the other. Transwell plates containing hAdMSCs cultured alone were used as controls.

#### Immunostaining and confocal microscopy

Immunocytochemical analysis for α-amylase (Santa Cruz, CA) was performed to evaluate the transdifferentiation of co-cultured hAdMSCs after 5 days of culture. Briefly, cells were fixed for 30 min in 4% PFA, washed three times with PBS, incubated for 1 h at room temperature in antibody diluent reagent solution, and incubated overnight at 4°C in a humid chamber with anti-α-amylase antibodies. After rinsing 3 times with PBS, slides were incubated at room temperature for 1 h with Alexa Fluor 488 Goat Anti-Mouse IgG (Molecular Probes, Invitrogen, Carlsbad, CA), washed three times in PBS, covered with Vectashield mounting medium containing DAPI, and analyzed under a fluorescence microscope.

#### Reverse transcription polymerase chain reaction

Total RNA was isolated from cells using TRIzol Reagent (Invitrogen) and converted to cDNA using the PrimeScript™ RT reagent Kit (TaKaRa, Otsu, Japan). The PCR amplification of α-AMY was performed using AccuPower PCR PreMix (Bioneer, Korea) at 94°C for 5 minutes, 30 amplification cycles (94°C for 30 seconds, 55°C for 30 seconds, and 72°C for 30 seconds), followed by extension at 72°C for 10 minutes. The primers used for the PCR were forward AATTGATCTGGGTGGTGAGC and reverse CTTATTTGG- CGCCATCGATG.

### Statistical Analysis

Statistical analysis was conducted using the Graph Pad Prism 5 package (GraphPad Software Inc., La Jolla, CA). The Mann-Whitney test was used to determine differences between two groups, and the Kruskal-Wallis test with Dunn’s post hoc test to determine differences between three groups. P values of less than 0.05 were considered statistically significant.

## Results

### Characteristics of hAdMSCs

The mesenchymal stem cells used in this study were highly purified with the CD73- and CD90-positive, and CD31-, CD34- and CD-45-negative immunophenotype. Multiple differentiation capacity towards osteogenic, adipogenic and chondrogenic lineages was also confirmed *in vitro* (Fig. S2). These cell characteristics are consistent with previous findings obtained using the same culture methods. [15].

### Macromorphologic and Functional Improvements after hAdMSC Administration

Macromorphologic findings at 12 weeks after irradiation showed that irradiation significantly reduced body weight in irradiated untreated mice (24.3±1.8 g, *P*<0.05) as compared with non-irradiated normal mice (27.5±2.8 g, Fig. S3A). Intravenously injected hAdMSCs tended to increase body weights in the hAdMSC-treated group (26.4±1.5 g) versus the irradiated group, although this increase was not statistically significant. However, SG weights at 12 weeks after irradiation were not significantly different among the three study groups (*P* = 0.255, Fig. S3B).

**Figure 1:**
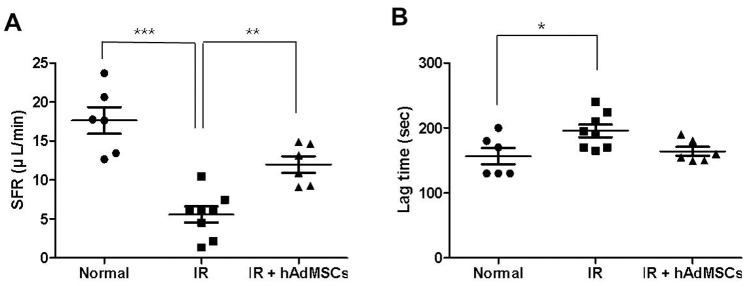
Macromorphologic findings at 12 weeks after irradiation. (A) Saliva production (SFR, µL/min) post-stimulation in the hAdMSC group was significantly higher than in the irradiated group. (B) The hAdMSC group tended to have shorter time to salivation (lag time). (^*^ *P*<0.05, ^**^ *P*<0.01, ^***^ *P*<0.001).

To determine whether hAdMSC administration improved salivary secretory function, saliva production rates and salivary lag times were measured at 12 weeks after irradiation. The irradiated group showed a significantly reduced ability to produce saliva (5.56±2.91 µg/min) as compared with the normal group (17.64±4.2 µg/min, *P*<0.001, Fig. 1A). SFR post-stimulation was significantly higher (11.98±2.6 µg/min) in the hAdMSC group than in the irradiated group (*P*<0.01). Salivary lag time was significantly greater in the irradiated group (195.5±27.42 sec, Fig. 1B) than in the normal group (156.7±30.77 sec, *P*<0.05). Salivary lag time in the hAdMSC group showed a trend toward improvement (164±17.03 sec), but this was not significant.

### Changes in the Micromorphologies and Functionalities of Transplanted Submandibular Glands

**Figure 2:**
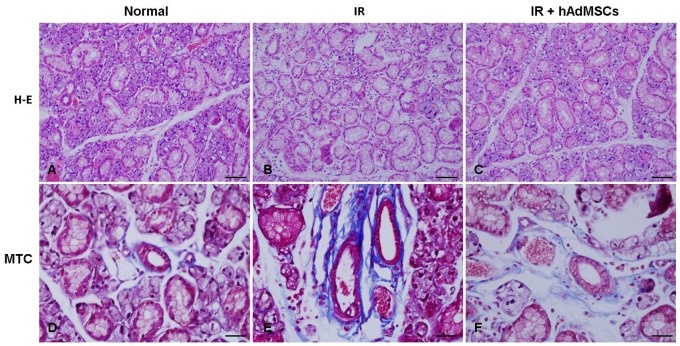
The micromorphologic changes after irradiation were visualized by H-E and MTC stainings at 12 weeks after irradiation. hAdMSC-treated SGs showed a well-preserved morphology and an greater number of acini than irradiated SGs. hAdMSC-treated SGs also exhibited less periductal and perivascular fibrosis than untreated SGs. Bar = 50 µm in H-E and 100 µm in MTC staining.

Microscopic morphologic changes were visualized by hematoxylin-eosin (H-E) or Masson’s trichrome (MTC) staining. H-E staining revealed at 12 weeks after irradiation, hAdMSC-treated SGs showed more preserved structures and greater numbers of acini than irradiated, untreated SGs (Fig. 2). hAdMSC-treated SGs also exhibited less periductal and perivascular fibrosis than untreated SGs.

**Figure 3:**
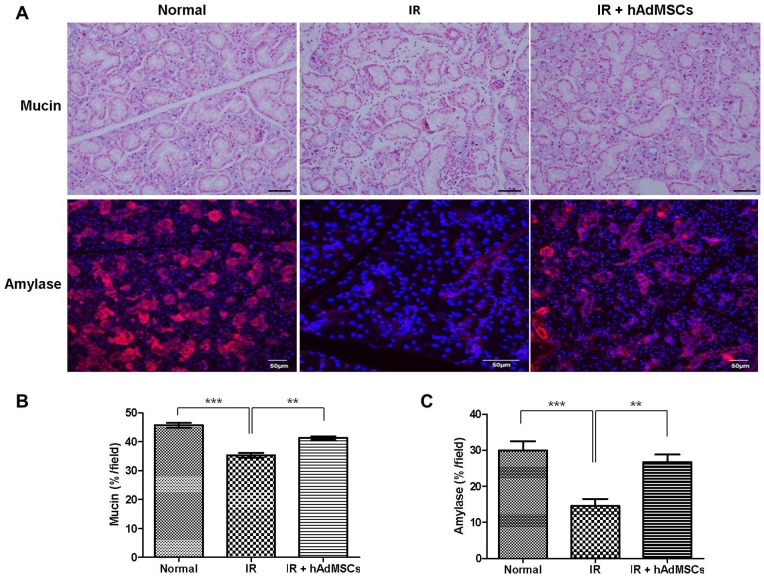
Functionalities of acinar cells at 12 weeks after irradiation. (A) The functions of acini after irradiation were evaluated by quantifying the surface areas occupied by mucin and amylase in salivary gland tissues. The productions of mucin (B) and amylase (C) were lower in irradiated SGs than in normal SGs. SGs in the hAdMSC group showed more mucin and tissue amylase production than SGs in the irradiated group. At least three random tissue sections per gland from ten mice were chosen for evaluation. Bar = 50 µm, ^**^ *P*<0.01, ^***^ *P*<0.001.

The functionalities of acini were assessed by measuring mucin and amylase production in SG acini (Fig. 3A). Mucin-containing acini stained with alcian blue (AB) appeared to be more numerous in hAdMSC-treated SGs than in irradiated SGs. These findings were confirmed by quantifying the surface areas occupied by mucin (*P*<0.01, Fig. 3B). Immunohistochemical analysis of SGs showed that amylase production was significantly lower in irradiated SGs than in normal SGs (*P*<0.001, Fig. 3C). However, SGs in the hAdMSC-treated group showed higher levels of tissue amylase production than SGs in the irradiated group, and this finding was confirmed by densitometric analysis (*P*<0.01).

### Protective Effect of hAdMSCs against Radiation-induced Salivary Tissue Damage

**Figure 4:**
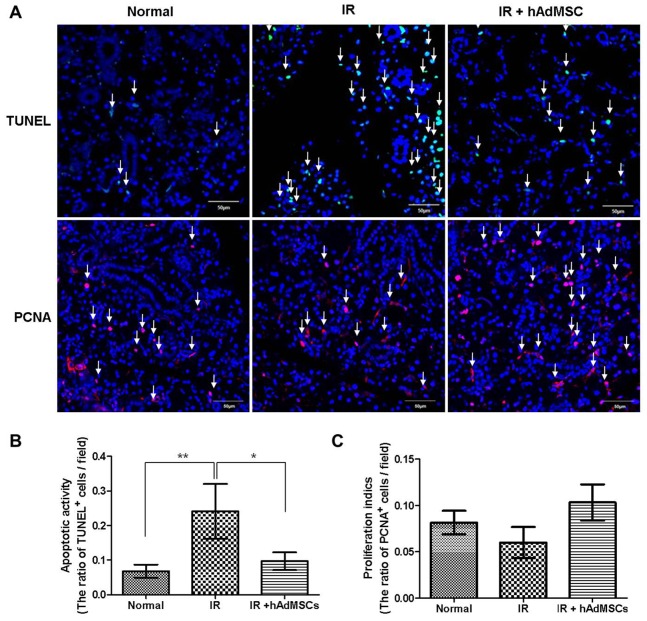
Immunofluorescent TUNEL and PCNA assays at 4 weeks after irradiation. (A and B) TUNEL assays revealed few TUNEL-positive apoptotic cells among normal SGs, but significant numbers in irradiated SGs. hAdMSC administration significantly reduced TUNEL-positive apoptotic cell numbers in the hAdMSC group as compared with the irradiated group. (A and C) PCNA assays conducted at 4 weeks after transplantation showed that proliferation indices tended to be higher for hAdMSC-treated SGs than for untreated SGs. The ratios of TUNEL- and PCNA-positive cells versus all DAPI-positive cells were calculated in random tissue sections. At least three random tissue sections per gland from ten mice were chosen for evaluation. Bar = 50 µm, ^*^ *P*<0.05, ^**^ *P*<0.01.

Next, we explored the mechanisms responsible for these morphological and functional improvements in hAdMSC-treated SGs after irradiation (Fig. 4A). A TUNEL assay at 4 weeks after irradiation revealed that few TUNEL-positive apoptotic cells were observed in normal SGs, whereas in irradiated SGs, the number of irradiation-induced apoptotic cells was significantly higher (*P*<0.01, Fig. 4B). hAdMSC administration significantly reduced the number of TUNEL-positive apoptotic cells as compared with the irradiated group (*P*<0.05).

PCNA assays conducted at 4 weeks after transplantation showed that proliferation indices tended to be higher for hAdMSC-treated SGs than for untreated SGs at 4 weeks after irradiation (Fig. 4C).

### Homing and the Transdifferentiation Potential of hAdMSCs after Systemic Administration

**Figure 5:**
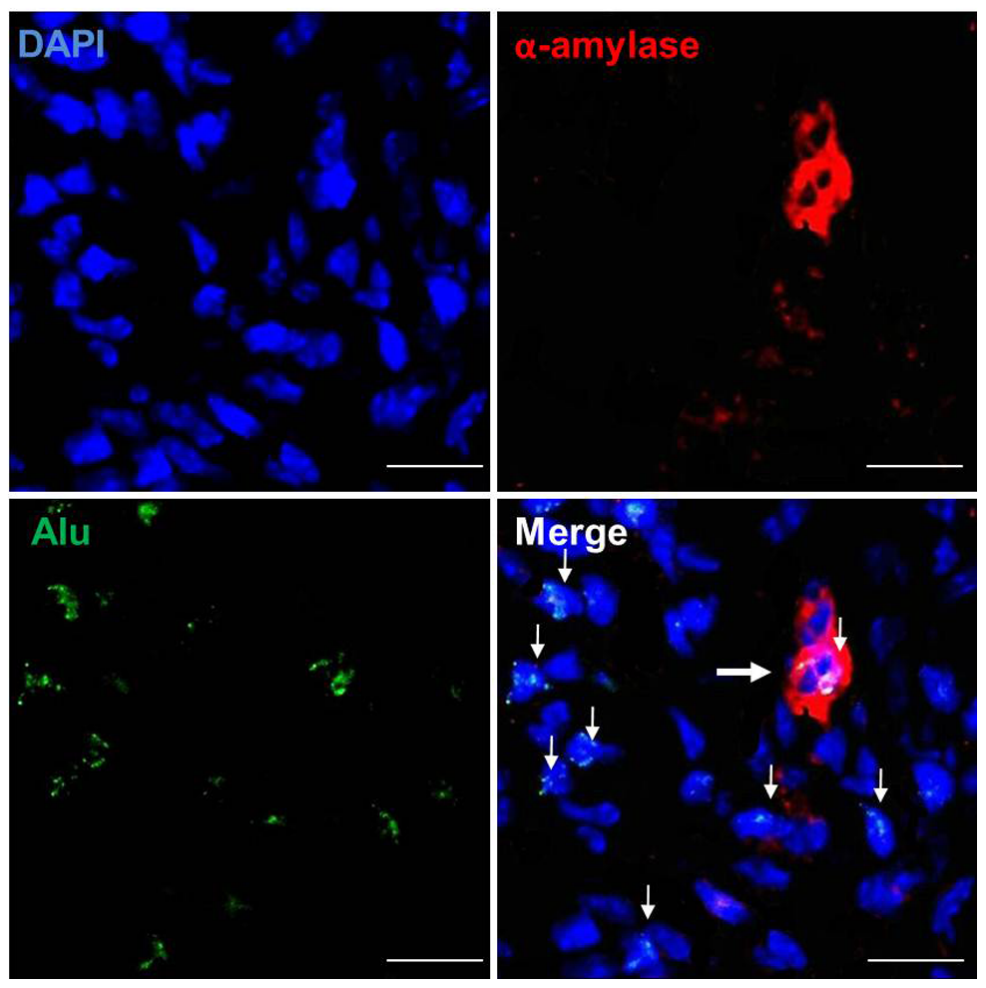
Homing of intravenously administered hAdMSCs to irradiated SGs and their transdifferentiation into SGCs at 4 weeks after systemic administration. FISH revealed cells reactive to human specific Alu DNA (green, arrow) in irradiated SGs. A few Alu-positive cells were also positive for the acinar cell marker, α-amylase (red). At least three random tissue sections per gland from ten mice were chosen for evaluation. DAPI (blue), Bar = 20 µm.

Next, we investigated whether intravenously administered hAdMSCs homed to irradiated SGs and transdifferentiated into SGCs. The presence of human-specific chromosomal DNA was analyzed by FISH using human Alu DNA. To verify the transdifferentiation of engrafted hAdMSCs, immunofluorescence histochemistry was performed using α-amylase (Fig. 5). At 4 weeks after systemic transplantation, hAdMSCs reactive to human-specific Alu by FISH were found in SGs damaged by irradiation, whereas no positive immunostaining was observed in control mice. Some human Alu-positive cells also expressed α-amylase, the acinar cell marker. These results suggest that systemically administered hAdMSCs are able to transdifferentiate into SGCs.

### *In vitro* Transdifferentiation of Cocultured hAdMSCs

**Figure 6:**
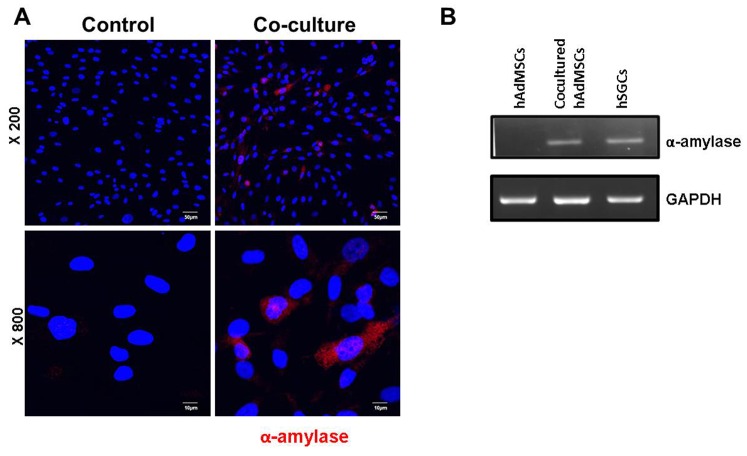
Immunofluorescent microscope image showing the transdifferentiation of hAdMSCs into amylase-producing cells after co-culture with SGCs. (A) Approximately 13%–18% of hAdMSCs co-cultured in the upper chamber of a Transwell co-culture system for 5 days stained positively for α-amylase (red). AdMSCs cultured alone only showed the nuclear expression of hAdMSCs (blue). Bar = 50 µm (B) Reverse transcription-polymerase chain reaction analysis confirmed α-AMY gene expression from the cocultured hAdMSCs, but no band was seen in the hAdMSC control. Human SGCs were used as a positive control.

Finally, we confirmed the *in vivo* finding that hAdMSCs transdifferentiated into SGCs using an *in vitro* co-culture system. The results obtained showed that a low number of co-cultured hAdMSCs (13%–18%) stained positively for α-amylase, whereas α-amylase staining was not observed in hAdMSCs cultured alone (Fig. 6A). α-AMY gene expression was detected in cocultured hAdMSCs, but was detected in control hAdMSCs (Fig. 6B). This observation suggests that hAdMSCs possess the potential to transdifferentiate into SGCs producing amylase *in vitro*.

## Discussion

This study was undertaken to determine whether the systemic administration of human adipose-derived mesenchymal stem cells (hAdMSCs) ameliorates radiation-induced SG damage. The findings obtained showed that intravenously infused hAdMSCs engraft into the SGs of mice subjected to local irradiation. In addition, it was found that hAdMSCs transplanted into irradiated SGs afford protection against radiation-induced cell damage, promote regeneration of SGCs, and some of localized cells have the potential to transdifferentiate into SGCs of the endodermal lineage.

The specific homing of systemically infused mesenchymal stem cells (MSCs) to irradiation-induced lesions was demonstrated in 2006 by Francois *et al*., who suggested the migration of MSCs to injured tissues is initiated by a signal released from inflamed tissue shortly after injury, and that level of MSC engraftment is related to the timing, dose, and distribution of irradiation. [17] Furthermore, it has been reported that human MSCs show persistent engraftment when transplanted into an immune-incompetent host. [18] The homing to injured SGs by administered cells can be also promoted using an enhancing agent. [11].

In this study, we administered hAdMSCs systemically within 6 hours of 15 Gy of neck irradiation and repeated the infusion once a week for 3 consecutive weeks. Infused hAdMSCs were found to have localized 4 weeks after initial infusion. However, immunofluorescent staining revealed the number of hAdMSCs that successfully engrafted and differentiated to SGCs was low. Although we did not perform quantitative analysis, MSC engraftment in damaged SGs was also observed by Sumita et al., who detected 9% donor-derived salivary epithelial cells in BSC-treated SGs after an intravenous infusion immediately after irradiation. [19] Additional investigations on the optimal number of delivered cells and on the optimal timing required for clinically useful damaged tissue restoration are required.

AdMSCs have been used for cellular therapy via local or systemic administration for a variety of indications. In particular, the systemic infusion of AdMSCs has been shown to be beneficial in the contexts of graft-versus-host-defense (GVHD), [20] rheumatic disease, [21] and thyroiditis. [22] AdMSCs appear to be capable of migrating to an injured site through the bloodstream and to have a positive effect on the repair or restoration of damaged tissues via several mechanisms, which include paracrine effects, vasculogenesis, cell fusion and/or cell transdifferentiation.

Recently, Kojima *et al.* reported that the direct administration of AdMSCs has the potential to restore SG function by restoring blood flow within submandibular gland tissues. [22] However, they did not observe significant transdifferentiation into salivary gland cells. In our experimental setup, the systemic administration of hAdMSCs after irradiation improved morphology and functional regeneration, not only in terms of protection against radiation-induced cell death but also because of the replacement of SGCs by transdifferentiation. Moreover, our coculture experiment revealed that cocultured hAdMSCs expressed α-amylase *in vitro,* and furthermore, α-AMY gene expression was also confirmed by PCR. These findings are in accord with earlier work published by Maria and Tran, [23] who found that a proportion of MSCs temporarily adopt a salivary epithelial phenotype by mesenchymal-to-epithelial transition induced by cross-talk between cells and the microenvironment. To the best of our knowledge, our results provide first evidence of the functional restoration of salivary glands by the systemic migration of hAdMSCs into irradiation-damaged tissues and of the contribution made by hAdMSCs to tissue repair by differentiation. These results also support the notion that MSCs derived from embryonic mesenchyme have the potential to differentiate into different tissue lineages, such as, hepatocytes and pancreatic cells. [24], [25].

The tissue regeneration of SGs has also been suggested to be related to AdMSC-induced paracrine stimulation. Recently, the release of paracrine mediators, such as, growth factors and chemokines, was reported to provide acute radioprotection. [26] In the present study, the reduction of apoptosis observed in the hAdMSC-treated SGs was probably related to the anti-apoptotic effects of stem cells due to the local paracrine secretion of hAdMSC-derived bioactive components. These findings are consistent with a previous report, in which it was found that BSC-transplanted SGs showed increased tissue regenerating activities, such as, blood vessel formation and cell proliferation, and a decrease in apoptotic activity. [19] In the present study, infused hAdMSCs survived at lesions for 4 weeks, but only a few transdifferentiated into salivary acinar cells, which produce amylase *in vivo*. This suggests that a paracrine effect, rather than transdifferentiation, is responsible for the amelioration of radiation damage by promoting the survival of endogenous progenitor cells or neovascularization. Further investigations are required to elucidate the process responsible for hAdMSC-mediated SG regeneration.

The efforts of researchers to establish the safety of MSC infusion have led to the use of MSCs delivered via systemic infusion for the treatment of various diseases. [27] We used human-derived AdMSCs cultured and expanded under GMP conditions, because this is for a prerequisite of future clinical trials. In fact, hAdMSCs have already been determined to be non-toxic and non-tumorigenic in animal models and in clinical trials. [15] In the present study, after systemic transplantation, hAdMSCs successfully engrafted in the xenogeneic SGs without signs of toxicity, and preserved their ability to undergo differentiation into mature tissue according to the recipient environment.

Xenogenic models are useful, but results are inevitably controversial. In the case of MSCs, although the xenogenicity does not interrupt the differentiation of MSCs, it can induce transplantation rejection. [17] Nevertheless, the survival of human MSCs after xenogeneic transplantation into immune competent mice without immunosuppression adjustment is presumed to be related to the immune-privileged properties of stem cells. [14] The immune evasion strategies of human MSCs have not been clarified, but several factors have been identified, including hypoimmunogenicity, the modulation of immune cell function, and the creation of a suppressive microenvironment. [28].

Several issues will need to be addressed in order to enable the clinical use of stem cell transplantation for the functional restoration of irradiation-inflicted SGs. First, a suitable preclinical animal model for transplantation that allows the engraftment of xenogeneic human stem cells should be established. Second, appropriate routes for transplantation, including intravenous injection, local transplantation by direct injection, and retroductal administration via an excretory duct, should be determined. Third, cultured cells should be harvested and isolated without contamination and expanded in a laboratory with adequate quality control. We believe our descripted model is useful for preclinical research purposes, and that it could be used to improve protection against radiation-induce damage and to restore salivary gland function after irradiation.

In conclusion, this study shows that systemically administered hAdMSCs are capable of migrating to an injured site through the bloodstream and becoming engrafted into xenogeneic SG tissues. Furthermore, our findings suggest hAdMSCs have the potential to transdifferentiate into SGCs and that they offer protection against radiation-induced cell damage. We believe that hAdMSCs should be viewed as candidates for cell-based therapy for the restoration of radiation-induced SG hypofunction.

## Supporting Information

Figure S1 **Schematic representation of the experimental setup.** The external irradiation dose of 15 Gy was administered to neck fields of C3H mice. Mice received 1×10^6^ hAdMSCs through the tail vein immediately after irradiation, and this infusion was repeated once a week for 3 consecutive weeks.
(TIF)

Figure S2 **Characterization of culture expanded human adipose mesenchymal stem cells.** (A) Flow cytometry analyses demonstrated that hASCs expressed CD105, HLA-ABC, CD44, CD29, CD73, and CD90, but were negative for HLA-DR, CD45, CD34, and CD31. (B) *In vitro* differentiation assays confirmed the multipotency of hASCs. Cells were positive for Alizarin red (upper-left), Oil Red-O (upper-middle), or Toluidine blue (upper-right) staining after osteogenic, adipogenic, or chondrogenic induction. Undifferentiated cells were not stained by Alizarin red or Oil Red-O staining (bottom). Scale bars = 100 µm.
(TIF)

Figure S3 **Macromorphologic findings at 12 weeks after irradiation.** (A) Locally irradiated mice showed significant decrease in body weights (BWs) measured at 12 weeks after irradiation. (B) Salivary gland weights (SGWs) normalized to BWs was not significantly different between the three study groups.
(TIF)
