# Supplementary figures and images for: Systemic Transplantation of Human Adipose Tissue-Derived Mesenchymal Stem Cells for the Regeneration of Irradiation-Induced Salivary Gland Damage
Source: PLoS One. 2013 Aug 9;8(8):e71167. doi: 10.1371/journal.pone.0071167 (PMC3739795; doi:10.1371/journal.pone.0071167)

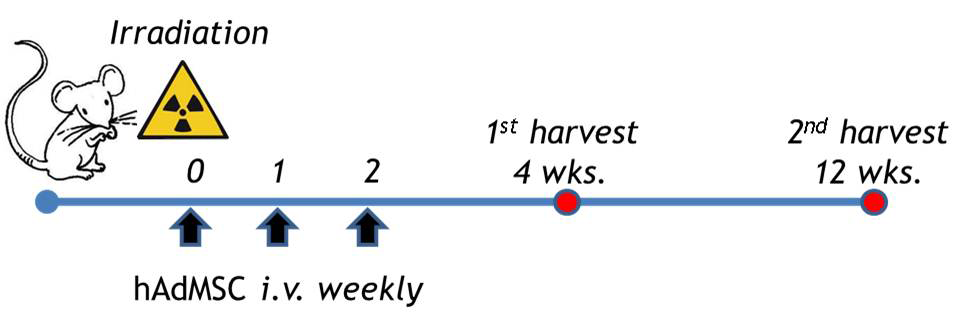

Supplement: Figure S1 — Schematic representation of the experimental setup. The external irradiation dose of 15 Gy was administered to neck fields of C3H mice. Mice received 1×10^6^ hAdMSCs through the tail vein immediately after irradiation, and this infusion was repeated once a week for 3 consecutive weeks. (TIF) [file pone.0071167.s001.tif]

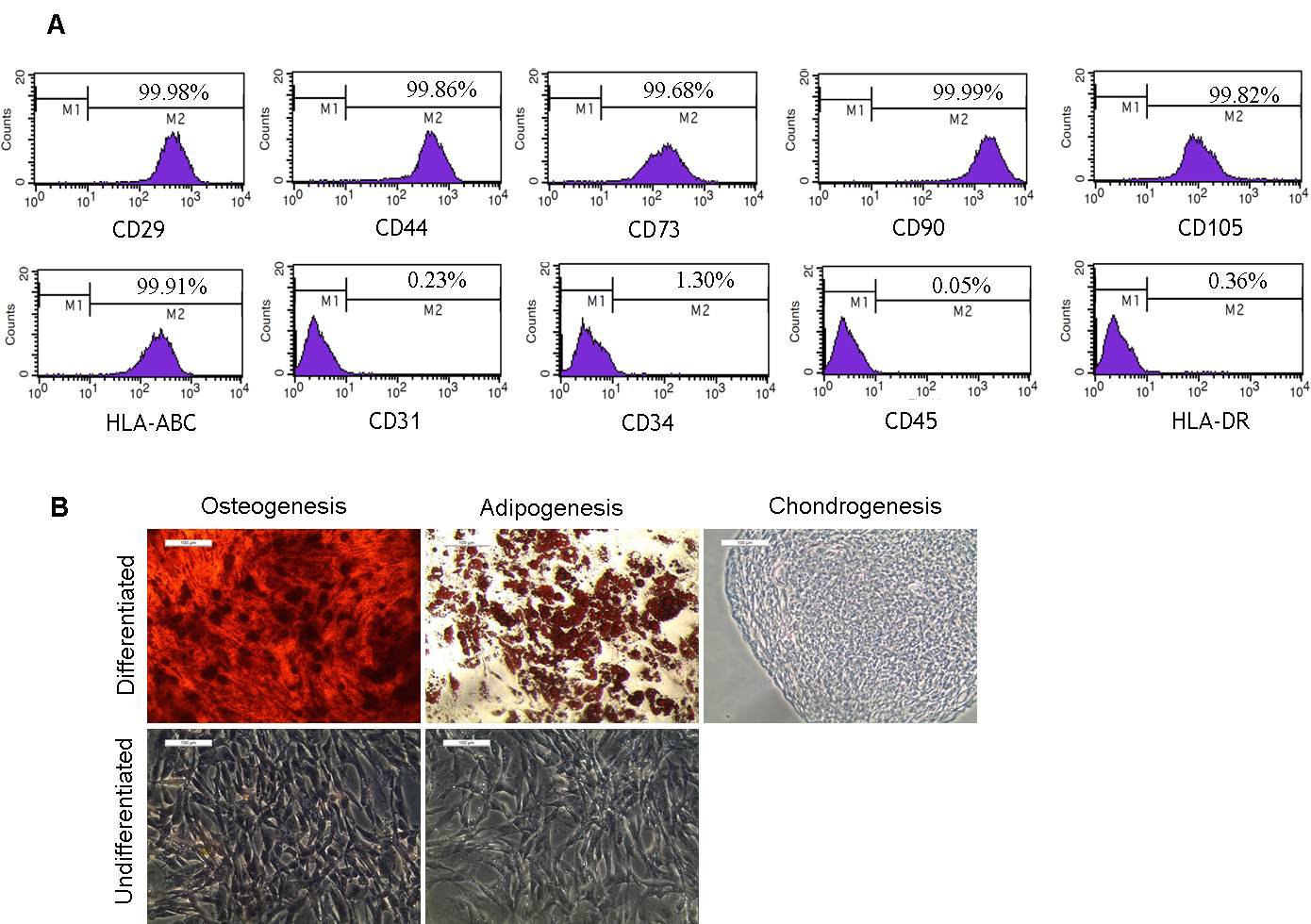

Supplement: Figure S2 — Characterization of culture expanded human adipose mesenchymal stem cells. (A) Flow cytometry analyses demonstrated that hASCs expressed CD105, HLA-ABC, CD44, CD29, CD73, and CD90, but were negative for HLA-DR, CD45, CD34, and CD31. (B) In vitro differentiation assays confirmed the multipotency of hASCs. Cells were positive for Alizarin red (upper-left), Oil Red-O (upper-middle), or Toluidine blue (upper-right) staining after osteogenic, adipogenic, or chondrogenic induction. Undifferentiated cells were not stained by Alizarin red or Oil Red-O staining (bottom). Scale bars = 100 µm. (TIF) [file pone.0071167.s002.tif]

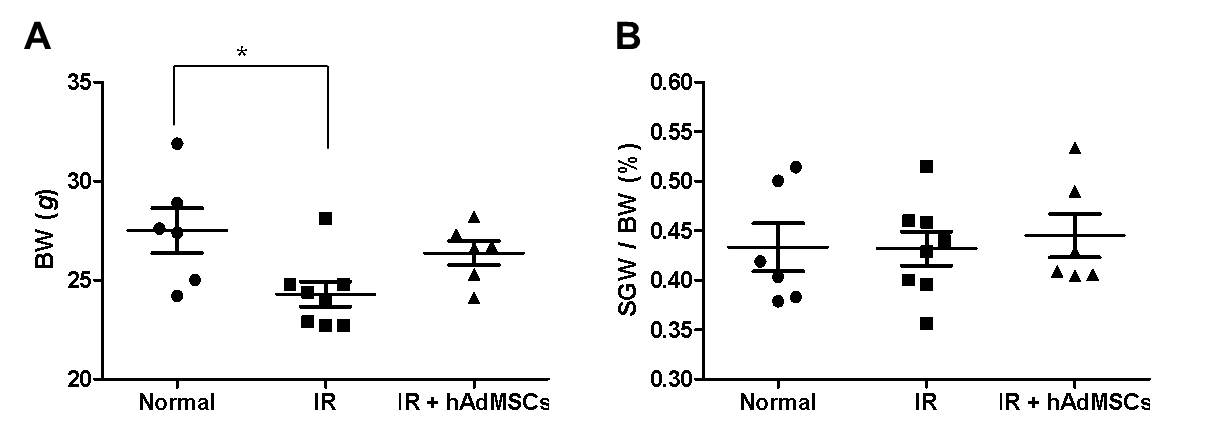

Supplement: Figure S3 — Macromorphologic findings at 12 weeks after irradiation. (A) Locally irradiated mice showed significant decrease in body weights (BWs) measured at 12 weeks after irradiation. (B) Salivary gland weights (SGWs) normalized to BWs was not significantly different between the three study groups. (TIF) [file pone.0071167.s003.tif]
